# Preparation and Performance Evaluation of Self-Cementing Nanoscale Polymeric Microspheres with Salt and Temperature Tolerance

**DOI:** 10.3390/molecules29112596

**Published:** 2024-05-31

**Authors:** Guohui Qu, Bowen Li, Yikun Liu, Zilu Zhang, Lifeng Bo, Jiqiang Zhi, Xuebin Tian, Xiaorui Bai, Xiunan Li, Qi Lv

**Affiliations:** 1 Key Laboratory of Enhanced Oil Recovery, Northeast Petroleum University, Ministry of Education, Daqing 163318, China; lbw714512@126.com (B.L.); liuyikun@nepu.edu.cn (Y.L.); zhijiqiang@nepu.edu.cn (J.Z.); 218003020722@stu.nepu.edu.cn (X.T.); 218003020671@nepu.edu.cn (X.B.); lxn990311@163.com (X.L.); lvqi11_28@163.com (Q.L.); 2 Taizhou Oil Production Plant of Sinopec East China Oil and Gas Branch Company, China Petroleum & Chemical Corporation, Taizhou 225300, China; zhanglaoban@stu.nepu.edu.cn; 3 Dongxin Oil Production Plant of Shengli Oilfield Company, China Petroleum & Chemical Corporation, Dongying 257000, China; bolifeng0926@stu.nepu.edu.cn

**Keywords:** temperature and salt resistance, the preparation of polymer microspheres, surfactant, performance evaluation, improving the recovery efficiency, microscopic displacement

## Abstract

Polymer microspheres with temperature and salt resistance were synthesized using the anti-suspension polymerization method, incorporating the functional monomers AMPS, AM, and AA. To enhance their self-gelling properties, the microspheres were designed with a core–shell structure. The shell is composed of a polymeric surfactant, fatty alcohol polyoxyethylene ether methacrylate (AEOMA), which serves as a thermosensitive crosslinking agent, enabling self-crosslinking upon shell decomposition, addressing compatibility with reservoir pore throat dimensions. Comprehensive characterizations including infrared spectroscopy, scanning electron microscopy, optical microscopy, and laser particle size analysis were conducted. The microspheres exhibited successful synthesis, a nanoscale size, and regular spherical morphology. They demonstrated excellent temperature and salt resistance, making them suitable for high-temperature, high-salinity reservoir profile control. With a stable three-dimensional network structure, the microspheres displayed good expansion behavior due to hydrophilic groups along the polymer chains, resulting in favorable water affinity. Even after aging, the microspheres maintained their gelling state with a distinct and stable microscopic network skeleton. They exhibited superior plugging performance in low-permeability reservoirs, while effectively improving water absorption profiles in reservoirs with permeability contrasts of 10 to 80, thereby enhancing oil recovery.

## 1. Introduction

After long-term water injection in oilfield development, the presence of reservoir heterogeneity and unfavorable fluid mobility ratios often leads to the formation of dominant channels in high-permeability reservoirs, resulting in inefficient or ineffective cycling in low-permeability reservoirs, thereby impacting overall field development and ultimate recovery [1,2,3,4,5]. Practice has shown that profile control technology has become an important means to improve water flooding in oilfield development, laying a solid foundation for stable production and increased oil recovery in domestic and international water-flooded reservoirs [6,7]. However, traditional profile control techniques only work near the wellbore, and as water continues to be injected, the injected water bypasses the blocked areas and re-enters the high-permeability zones [8]. Therefore, the concept of deep profile control and water blocking technology has been proposed, and with a deeper understanding of reservoirs, the application of chemicals in the treatment of deep reservoirs has gained widespread attention. Various deep profile control agents have been developed, including pre-crosslinked gel particles (PPG), polymer microspheres, inorganic gel coatings, and other agents [9,10,11,12,13]. Polymer microspheres are renowned for their excellent deep migration capability. They swell upon water absorption after injection into the reservoir and effectively block high-permeability channels through adsorption or bridging, allowing subsequent injected fluids to enter the medium-to-low-permeability layers, thus improving the reservoir’s water absorption profile [14,15,16,17,18,19]. Polymer microspheres also possess good deformability, making them easy to inject and capable of migrating deep into the reservoir, where they continue to exhibit their efficient blocking and profile control effects, thereby enhancing the recovery efficiency of heterogeneous reservoirs [20,21,22]. In some oilfields, not only are the reservoirs deep, but they also experience high temperatures (high-temperature conditions) and utilize produced water reinjection, resulting in high salinity of the formation water (high-salinity conditions). This poses challenges for conventional polymer microspheres, as their hydration and swelling performance are limited under high-temperature and high-salinity conditions. These microspheres are unable to effectively gel and tend to undergo dehydration and degradation, leading to poor long-term stability and inability to meet the requirements of deep-profile control in the reservoir. Many researchers have attempted to address this issue by introducing heat-resistant monomers into the copolymerization of acrylamide (AM) to prepare heat-resistant polymer microspheres. Lou et al. demonstrated that adding AMPS to the YG370-1 composition allows microspheres to maintain thermal stability at temperatures below 105 °C [23]. Zhu synthesized emulsion microspheres by incorporating AMPS and NVP into AM. These microspheres remained stable for 60 days in formation water with a concentration of 20 × 10^4^ mg/L at 90 °C; they then showed slight deformation but retained their spherical structure for up to 120 days [24]. Fethi et al. and Roussennac et al. reported that microspheres copolymerized from AM, acrylic acid (AA), temperature-resistant monomer AMPS, and styrene remained stable for over 30 days at 99 °C [25,26]. Lin et al. prepared terpolymer microspheres using two heat-resistant monomers, AMPS and NVP, with AM as the primary reagent. Their experiments indicated that the terpolymer microsphere/water dispersion system could withstand a temperature of 120 °C for 19 days [27]. These studies primarily focused on the thermal resistance of polymer microspheres, neglecting their stability under high-salinity conditions and their profile control capabilities under reservoir conditions. Additionally, many scholars have investigated the compatibility of microspheres with reservoir throat sizes [28]. Li examined the compatibility between polymer microspheres and reservoir pores using matching factors and permeability limits [29]. Zhao et al. and Dai et al. used matching factors to describe the fit between polymer microspheres and core pores, finding that polymer microspheres achieved optimal plugging and deep fluid diversion effects only within a specific range [30,31]. Current research predominantly discusses the matching of particle size with throat dimensions. The transport and retention of conventional polymer microspheres in the reservoir heavily rely on the matching between microsphere size and pore throat dimensions. If the microsphere size is too large or the swelling ratio is insufficient, it can lead to injection and channeling issues. Therefore, the focus in the field of profile control and water blocking using polymer microspheres should be on improving their temperature and salt resistance capabilities and enhancing their compatibility with the pore size distribution in the reservoir.

In response to the conditions in high-temperature and high-salinity oilfield reservoirs, a novel self-gelling polymer microsphere system with temperature and salt resistance has been developed and prepared. These polymer microspheres possess unique surface effects, system effects, and functional groups [21]. From a molecular perspective, the introduction of functional monomers or groups on the main chain and side chains can significantly improve the temperature and salt resistance of the polymer [32,33,34]. Acrylamide and acrylic acid copolymers were selected as monomers on the main chain to enhance temperature resistance, effectively avoiding thermal loss of microsphere particles in high-temperature environments. The presence of AMPS on the side chains enhances the salt resistance of the microspheres. Additionally, the presence of dimethyl groups in AMPS introduces steric hindrance to the acrylamide groups during the polymerization process, enhancing the rigidity of the copolymer. The crosslinking agent MBA is used to increase shear resistance, allowing the crosslinked microspheres to maintain a three-dimensional network structure and prevent shear-induced rupture during deep migration. The hydrophilic groups, including amide, sulfonic acid, hydroxyl, and ester groups, along the polymer chains form hydrogen bonds or chemical bonds with water molecules, resulting in bound water and enhancing the swelling performance of the microspheres in high-salinity environments [29,35,36]. The microspheres also possess a core–shell structure. For the initial crosslinking of the shell, a polymeric surfactant, fatty alcohol polyethylene glycol ether methacrylate (AEOMA), was employed as a thermosensitive crosslinking agent. This allowed for secondary gelation between the long polymer chains and divinylbenzene (DVB) upon shell decomposition. This secondary gelation facilitates particle size enlargement without solely relying on its own expansion properties, thereby improving the matching between the microspheres and the pore throat size of the reservoir.

This study employed a redox-initiated system and a reverse microemulsion polymerization method [37] to prepare temperature- and salt-resistant (AM-AA-AMPS) ternary polymer microspheres with a stable three-dimensional network structure. The monomers AM, AA, AMPS, and the crosslinking agent N,N′-methylenebisacrylamide (MBA) as well as poly (ethylene glycol) diacrylate (PEGDA) were used in the synthesis. The temperature resistance, salt resistance, swelling performance, stability, plugging performance, and micro-scale pore throat transport-plugging characteristics of the temperature- and salt-resistant self-gelling polymer microspheres were investigated [38,39,40,41]. These findings provide robust evidence for the implementation of temperature- and salt-resistant self-gelling nanoscale polymer microspheres for profile modification in high-temperature and high-salinity reservoirs.

## 2. Results and Analysis

### 2.1. Preparation and Characterization of Nano-Sized Polymer Microspheres

#### 2.1.1. Selection of Core Monomer of Polymer Microspheres

**Figure 1:**
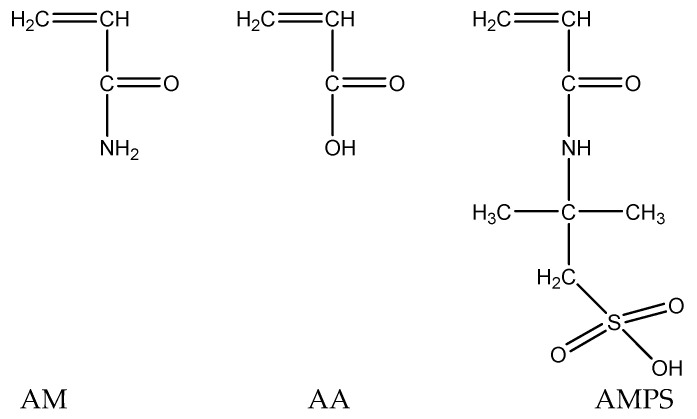
Selection of microsphere core monomer.

The fundamental factor influencing the temperature and salt resistance of nanoscale polymer microspheres is the molecular structure of the polymer. Different molecular structures exhibit different conformations under varying conditions. Based on the chemical mechanisms of temperature and salt resistance, along with economic considerations, the following main chain monomers have been selected, as shown in Figure 1.

To enhance temperature resistance, the monomer acrylamide (AM) and acrylic acid (AA) copolymers are chosen for the main chain, effectively avoiding thermal losses of microsphere particles in high-temperature environments. For the side chain, a water-soluble anionic surfactant monomer, 2-acrylamido-2-methylpropane sulfonic acid (AMPS), is selected to enhance salt resistance of the microspheres. The presence of dimethyl groups in AMPS creates steric hindrance during the polymerization process, enhancing the rigidity of the copolymer. The strong polarity of the SO_3_H group in AMPS promotes electrostatic repulsion and helps maintain the polymer in an extended state, making it less sensitive to salts. When interacting with divalent metal ions such as Ca^2+^ and Mg^2+^, the polymer is less prone to molecular chain curling and precipitation. Additionally, the bulky side group of AMPS enhances steric hindrance between polymer groups, increases chain rigidity, and improves the conformation of the polymer in solution. AMPS is easily polymerizable and widely available, making it a suitable functional monomer for temperature and salt resistance.

#### 2.1.2. Selection of Polymer Microsphere Shell Monomer

**Figure 2:**
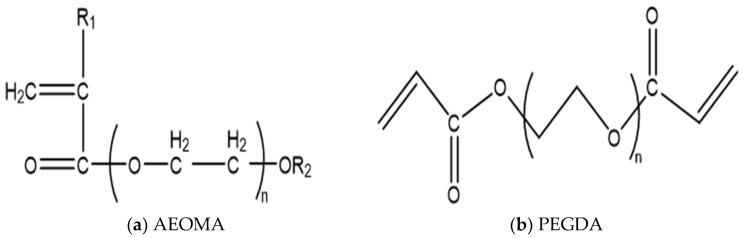
Selection of microsphere shell monomer. (**a**) Fatty alcohol polyoxyethylene ether methacrylate; (**b**) polyethylene glycol diacrylate.

The monomers chosen for the microsphere shell are acrylamide, acrylic acid (AA), and alkyl polyoxyethylene methacrylate (AEOMA). The crosslinking agent selected is polyethylene glycol diacrylate (PEGDA), with its molecular formula shown in Figure 2.

#### 2.1.3. Preparation of Nano-Scale Polymer Microspheres

**Figure 3:**
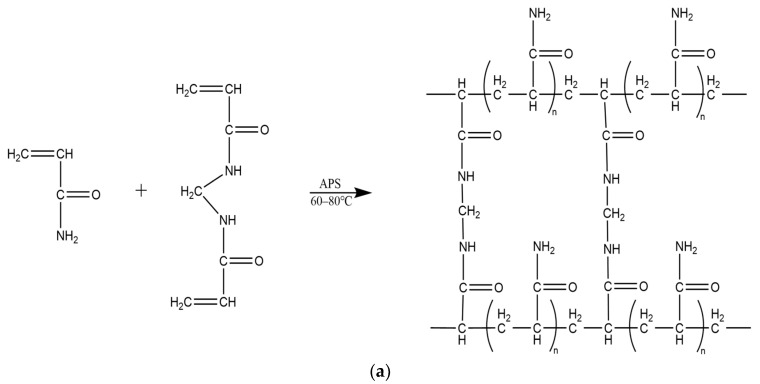

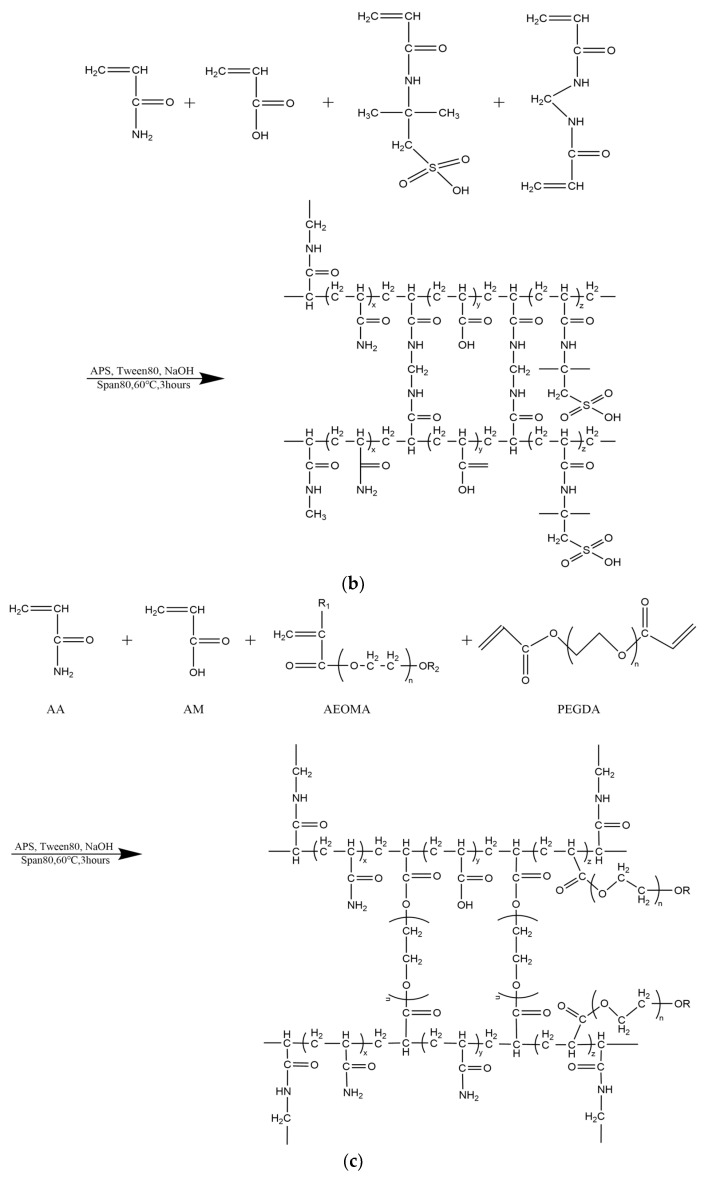
Preparation of polymer microspheres by reversed-phase emulsion polymerization. (**a**) Conventional polyacrylamide microsphere synthesis process and molecular structure. (**b**) Synthesis process and molecular structure of temperature- and salt-resistant self-gelatinizing microsphere cores. (**c**) Synthesis process and molecular structure of temperature- and salt-resistant self-gelatinizing microsphere shells.

Conventional microspheres are typically prepared by mixing acrylamide with a crosslinking agent, N,N′-methylenebisacrylamide, and an initiator, ammonium persulfate [42]. The mixture is then combined with the oil phase and maintained at temperatures ranging from 60 °C to 80 °C. After a half-hour waiting period, the solution is cooled, followed by the removal of the oil phase, resulting in the final production of nanospheres (microspheres) as depicted in Figure 3a.

**Figure 4:**
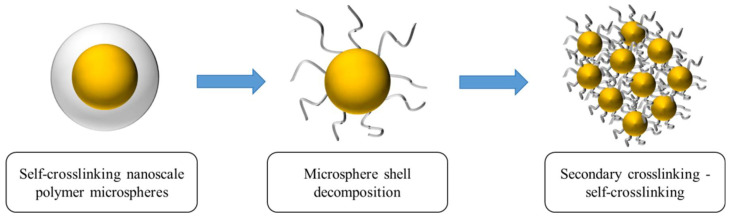
Schematic diagram of microsphere shells that can produce cementation after disintegration.

Temperature- and salt-resistant self-gelling nanoscale polymer microspheres were prepared using a reverse microemulsion polymerization method, as depicted in Figure 3b,c, which illustrates the reaction process and molecular structure. To enhance the self-gelling performance, a dual crosslinking structure and an intermittent feeding process were introduced, giving the microspheres a distinctive core–shell structure. After the decomposition of the shell, a gelling effect is generated, as shown in Figure 4.

(1) At room temperature, 36 g of Span80 is weighed and added to 70 mL of paraffin oil. The mixture is mechanically stirred at a stirring speed of 150–160 r/min until a uniform solution is obtained, resulting in the oil-phase liquid.
(2) In 25 g of water, 10 g of acrylamide (AM), 2 g of acrylic acid (AA), 3 g of 2-acrylamido-2-methyl propane sulfonic acid (AMPS), 0.045 g of N,N′-methylenebisacrylamide (MBA), and 3.2 g of TWEEN80 (polyoxyethylene sorbitan monooleate) are dissolved, and the pH is adjusted to neutral to obtain the core solution. In another 12 g of water, 5 g of acrylamide (AM), 1 g of acrylic acid (AA), 0.1 g of fatty alcohol polyethylene glycol ether methacrylate, and 0.06 g of polyethylene glycol diacrylate (PEGDA) are dissolved, and the pH is adjusted to neutral. Subsequently, 0.1 g of chromium acetate (DVB) is added to the solution and fully dissolved to obtain the shell solution.
(3) The oil-phase liquid is added to a three-neck flask and mechanically stirred at 800 r/min. Under these conditions, 4/5 of the core solution is added dropwise. After the completion of the dropwise addition, stirring is continued for an additional 30 min. The stirring speed is then reduced to 300 r/min, and nitrogen gas is purged into the three-neck flask for 30 min. Subsequently, the temperature is raised to 60 °C, and 1.2 mL of a 1% ammonium persulfate (APS) solution is added dropwise to initiate the reaction. After 40 min, the remaining 1/5 of the core solution is added dropwise to the three-neck flask. Upon completion of the core solution addition, the shell solution is added dropwise to the three-neck flask. After the complete addition of the shell solution, 0.5 mL of a 1% APS solution is added dropwise. The polymerization reaction is allowed to proceed for 6 h, and polymer microspheres are obtained.

#### 2.1.4. Structural Characterization of Nano-Sized Polymer Microspheres

(1) Infrared spectrum characterization of polymer microspheres

**Figure 5:**
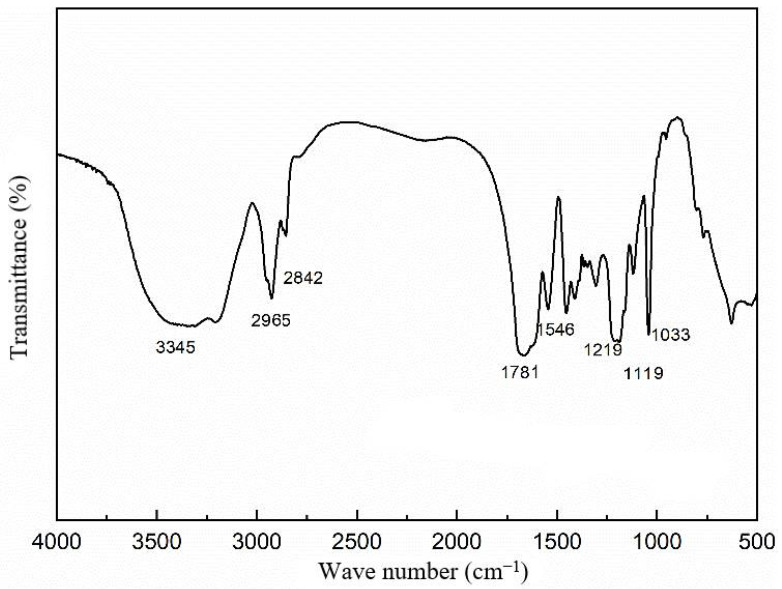
Infrared spectra of polymer microsphere particles.

Figure 5 and Table 1 present the infrared spectroscopy graph and the analysis of infrared data for the purified microsphere particles.

Based on the experimental results shown in Table 1, several absorption peaks can be identified. The peak at 3345 cm^−1^ corresponds to the stretching vibration of N-H in the amide group (-CONH). The peaks at 2965 cm^−1^ and 2842 cm^−1^ are attributed to the stretching vibrations of C-H in the saturated hydroxyl groups -CH_2_ and -CH_3_, respectively. The peak at 1781 cm^−1^ represents the characteristic absorption of the -C=O bond in the amide group (-CONH). The peak at 1542 cm^−1^ corresponds to the characteristic absorption of the -C=O bond in the carboxyl group (-COO-). The peaks at 1219 cm^−1^, 1119 cm^−1^, and 1033 cm^−1^ are associated with the in-plane bending vibrations of S=O in the -SO_3_^−^ group. Notably, no absorption peak attributed to C-H in the saturated carbon–carbon double bond is observed, indicating the cleavage of the double bond in the monomer. The infrared spectrum confirms the thorough polymerization of the monomer, resulting in the formation of the desired product.

(2) Morphology of polymer microspheres

**Figure 6:**
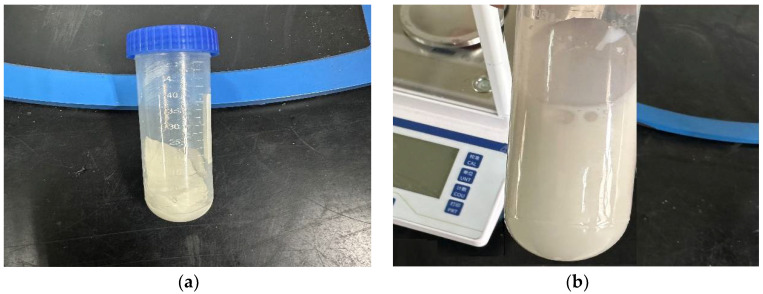
Appearance morphology of polymer microsphere particles. (**a**) Microsphere dry powder; (**b**) microsphere emulsion.

The visual observation of the dry powder appearance of the microspheres is depicted in Figure 6a. It can be seen that the macroscopic morphology of the microsphere particles appears as white powder-like solids. Similarly, the visual observation of the microsphere emulsion solution is shown in Figure 6b. The emulsion appears as a white liquid and exhibits complete dispersion in water, without any flocculent or precipitate formation.

**Figure 7:**
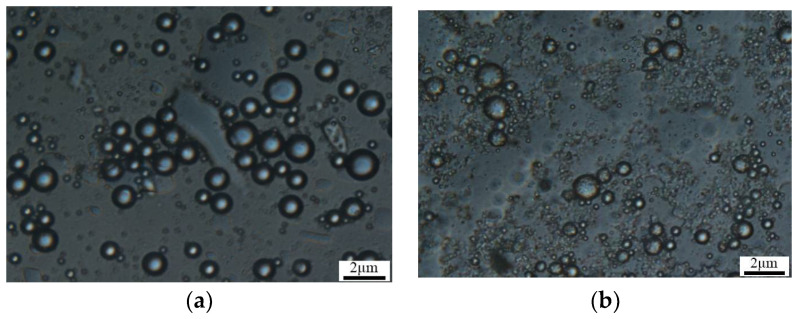
Two groups of polymer microsphere microscopes. (**a**) Sample 1; (**b**) sample 2.

The microsphere’s microscopic morphology under an optical microscope is shown in Figure 7. By dispersing the microsphere powder obtained from different experimental batches in a liquid medium, it can be observed that both groups of microspheres are relatively well dispersed in water and do not agglomerate after stirring for 30 min. This is advantageous for the injection of the microsphere solution. The microspheres exhibit a uniform spherical shape, indicating that the experimentally synthesized microspheres have a relatively narrow size distribution, with sizes of approximately 400 nm. The synthesized product meets the required specifications.

**Figure 8:**
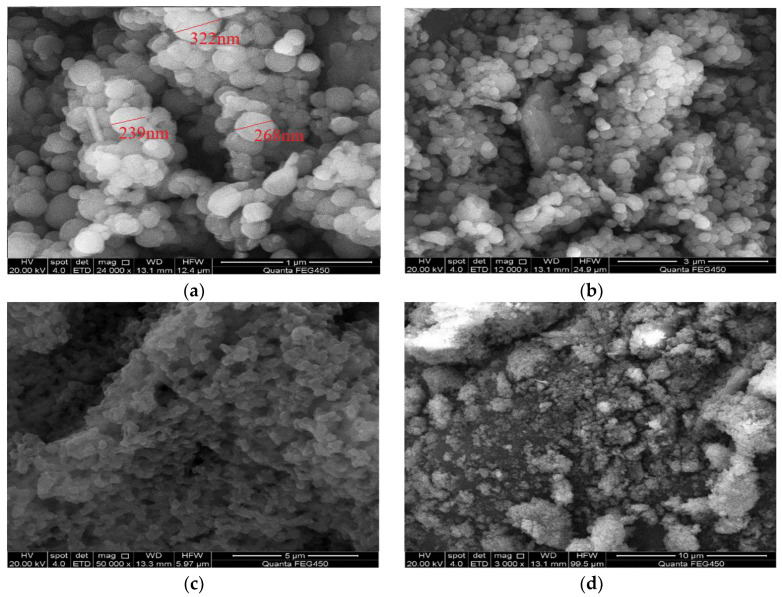
Scanning electron microscopy of polymer microspheres. (**a**) 1 μm; (**b**) 3 μm; (**c**) 5 μm; (**d**) 10 μm.

The morphology of the polymer microsphere powder under a scanning electron microscope is shown in Figure 8. From the image, it can be observed that the polymer microspheres are uniformly spherical with a consistent particle size distribution, and the particle size is on the nanometer scale.

The microspheres exhibit high integrity with a smooth and pore-free surface, which is advantageous for injection into the reservoir. They possess good injectability, effectively reducing the cost and difficulty of injection operations. The uniform and crack-free surface of the microspheres also facilitates their migration deep into the reservoir. They play a plugging role in the deep areas of high-permeability reservoirs, thereby improving reservoir heterogeneity and enhancing oil recovery. When observing the microsphere’s microscopic morphology at different magnifications, it can be observed that the microsphere powder shows some agglomeration with adhesion between microspheres. In high-permeability reservoirs, they can self-aggregate and form strong plugging characteristics. To better analyze the performance of the polymer microspheres, it is necessary to measure their overall particle size distribution.

(3) Particle size distribution of polymer microspheres

**Figure 9:**
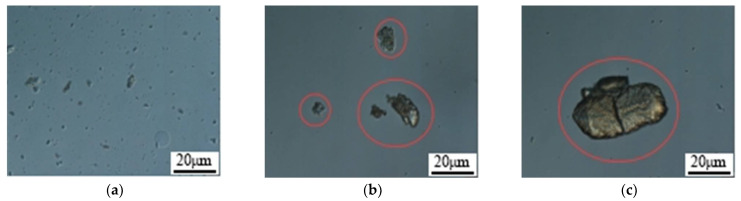
Optical microscopic observation of microspheres under different dispersants. (**a**) Nanospheres—reinjection water; (**b**) nanospheres—anhydrous ethanol; (**c**) nanospheres—kerosene.

Due to the phenomenon of agglomeration in polymer microspheres, the measurement of particle size often leads to inaccurate results. To address this issue, a certain amount of the same polymer microsphere dry powder is taken and placed in different dispersing liquids (20,000 mg/L salinity injection water, ethanol, and white oil) for testing. The dispersion of the microspheres is observed using an optical microscope, as shown in Figure 9.

According to Table 2, the dispersion of polymer microspheres in the injection water solution is good, showing no significant agglomeration or bonding. However, agglomeration is evident in the ethanol and kerosene solutions. Therefore, it is recommended to continue using injection water as the dispersing agent for the measurement of polymer microsphere particle size.

**Figure 10:**
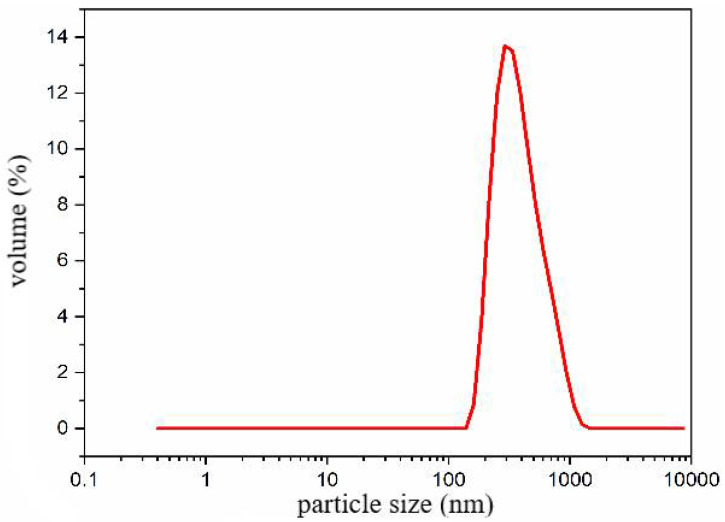
Nano-scale plugging agent dynamic light scattering test particle size distribution.

Therefore, a microsphere solution with a concentration of 3 wt% was prepared using microsphere powder and field backflow water with a salinity of 20,000 mg/L. The particle size distribution of the solution was measured at room temperature (25 °C) using a laser particle size analyzer, and the results are presented in Figure 10. The particle size distribution of the polymer microspheres mainly ranged from 110 to 900 nm (D0.1–D0.9), with a median particle size (D0.5) of 407 nm.

### 2.2. Evaluation of Physical and Chemical Properties of Polymer Microspheres

#### 2.2.1. Evaluation of Temperature Resistance

The evaluation of thermal stability using thermogravimetric analysis (TGA) is crucial for assessing the temperature resistance of polymer materials. At elevated environmental temperatures, various reactions occur within the polymer material. Initially, small molecules and side chains experience breakage. As the temperature increases, the main polymer chains start to break, leading to a significant disintegration of the polymer material and a subsequent decline in its performance. Therefore, thermal stability is an important indicator for evaluating the performance of polymer materials.

**Figure 11:**
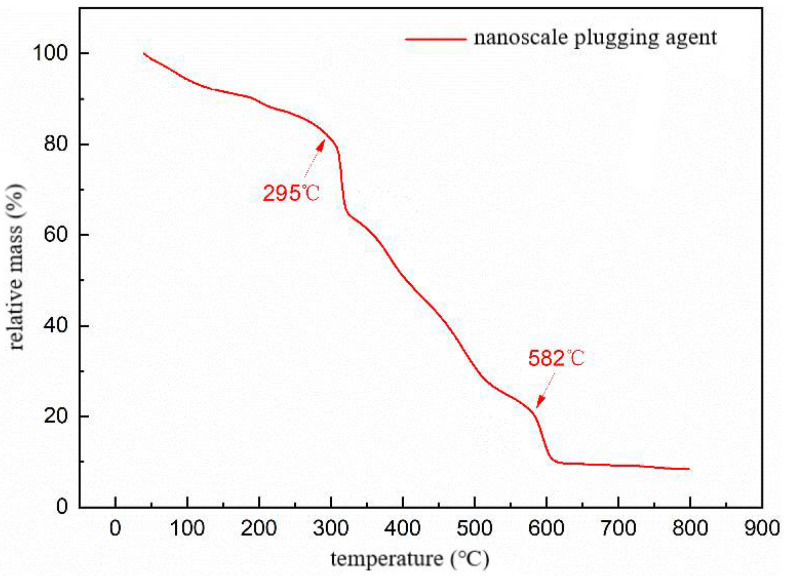
TG curve of polymer microsphere particles.

In oilfield applications, polymer microspheres are used for deep profile control and water flooding. The reservoir temperatures in oilfields are often high, requiring polymer microspheres to have intact molecular chains and excellent thermal stability to effectively perform plugging and profile control functions. Here, the nanoscale polymer microspheres obtained from experiments were evaluated using thermogravimetric analysis. The experimental results are shown in Figure 11.

Figure 11 depicts the thermogravimetric curve of the polymer microsphere particles, which exhibits three distinct stages of thermal weight loss. The first stage occurs from 25 to 295 °C, during which intermolecular and intramolecular water evaporation takes place, with a weight loss of 19%. The second stage occurs between 295 and 582 °C and involves the imidization reaction of amide groups and the decomposition of side chains such as AMPS (2-acrylamido-2-methylpropane sulfonic acid), with a weight loss of 60.5%. The third stage occurs above 582 °C and is primarily attributed to carbonization, main chain rupture, and decomposition of the polymer, with a weight loss of 11.5%. The total weight loss across the three stages reaches 88.5%. Overall, significant mass loss of the microspheres is observed only after the temperature exceeds 295 °C, indicating excellent thermal resistance of the polymer microspheres. This suggests that the microspheres exhibit good temperature stability and can be effectively utilized for reservoir profile modification under the high-temperature conditions of the field without undergoing thermal decomposition and weight loss.

#### 2.2.2. Evaluation of Salt Tolerance

Due to the inhibitory effect of high-salinity formation water in oilfields, the salt resistance of polymer profile control agents is particularly important. The experimental results are presented in Table 3, illustrating the salt resistance of the polymer microsphere particles.

According to Table 3, the initial particle size of the microspheres extracted in a dimethyl ketone solution is 407 nm. When preparing microsphere solutions using water with different salinity levels, the range of initial particle sizes is 510 nm to 410 nm. The microsphere emulsion prepared in pure water has the largest initial particle size, measuring 510 nm. In water with a salinity of 25,000 mg/L, the initial particle size is 410 nm, indicating that high-salinity water has a certain inhibitory effect on microsphere particle size growth. After 15 days of hydration, it can be observed that the particle sizes of the microspheres are similar under different salinity conditions. After 30 days of hydration, the microspheres show no degradation, and the particle sizes remain within a certain range. This is attributed to the introduction of the anti-salt functional monomer AMPS during the preparation of the polymer microspheres. The -SO_3_H group in AMPS exhibits tolerance to ions such as Na^+^, Mg^2+^, Cl^−^, and Ca^2+^ in high-salinity water, preventing the formation of precipitates through polymer–electrolyte crosslinking. Additionally, the presence of amide groups on the microsphere molecular chains reduces the structural impact of electrolytes. As a result, the polymer microsphere particles exhibit good salt resistance.

#### 2.2.3. Evaluation of Expansion Performance

The swellability performance is also an important criterion for evaluating the qualification of polymer microsphere particle preparation. In heterogeneous reservoir pore throats, nanoscale microspheres increase their volume by absorbing water and swell, thereby plugging high-permeability channels. This serves the purpose of water blocking and profile control.

(1) The effect of different salinity on the expansion properties of microspheres

**Figure 12:**
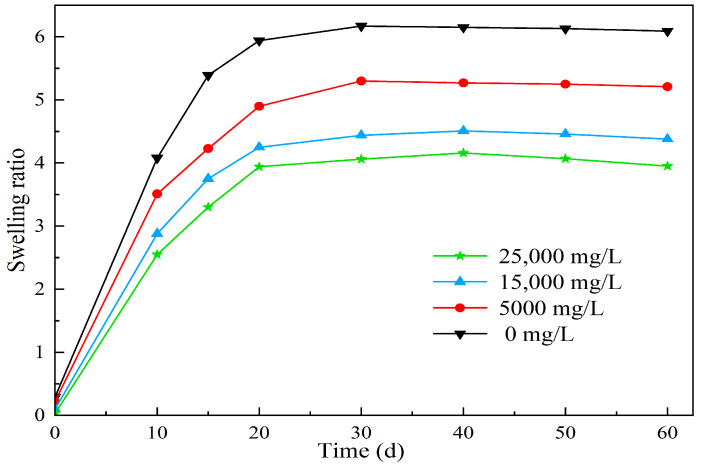
The effect of different salinity on the swelling properties of polymer microspheres.

Four oilfield injection water samples with salinity concentrations of 0 mg/L, 5000 mg/L, 15,000 mg/L, and 20,000 mg/L were used to prepare microsphere solutions. The volume of each solution was 100 mL, and the concentration of the microspheres was 3000 mg/L. The solutions were maintained at a temperature of 25 °C (room temperature) for 60 days. At different time intervals, a small amount of solution was extracted to measure the particle size and calculate the swell factor of the microspheres. The calculation results are shown in Table 4 and Figure 12.

From Figure 12, it can be observed that under simulated reservoir temperature conditions, the swell factor of polymer microspheres in different salinity solutions increases rapidly in the initial stage as the swelling days increase. However, the swell factor reaches equilibrium within the seventh to fifteenth day of swelling. This can be explained by the swelling mechanism of the microspheres. When the microspheres interact with water molecules, a solvation layer is formed. The hydrophilic groups such as amide groups, sulfonic acid groups, hydroxyl groups, and ester groups present in the polymer chains form hydrogen bonds or chemical bonds with water molecules, resulting in the formation of bound water. This process occurs rapidly and is accompanied by a thermal effect. Once the solvation layer is formed, the polymer chains extend and expand, leading to an increase in the microsphere volume. Simultaneously, some hydrophilic groups on the polymer chains undergo hydrolysis, resulting in the formation of mobile ions. This leads to higher ion concentration inside the three-dimensional network structure of the microspheres compared to the external concentration. Under the influence of osmotic pressure, water molecules enter the microspheres, further enhancing their swelling capacity. The internal crosslinked network structure of the microspheres also has water absorption capacity due to capillary effects. With the addition of free water, the long polymer chains inside the microspheres further extend and dissociate, promoting water absorption and swelling of the microspheres. As water absorption reaches a certain degree, the difference in osmotic pressure decreases, and the swelling rate slows down until it reaches equilibrium.

Furthermore, as shown in Figure 12, the expansion ratio of the microspheres gradually decreases with increasing salinity. The experimental results are consistent with the Flory theory. This phenomenon occurs due to the high content of anions and cations in high-salinity water. According to the free energy theory, after aging and swelling, the polymer microballs have numerous dissociable groups on their molecular chains, which can generate many positively charged cations and high molecular weight anions. The cations are randomly dispersed around the anions, forming a stable electric field. As the salinity increases, the number of surrounding cations also increases. Due to the shielding effect of the cations on the negative charges, the intermolecular forces of the polymer are weakened, reducing the elastic free energy of the system and making it more prone to stabilization. Consequently, the water absorption and swelling capacity of the polymer microballs decreases. Due to the presence of salt-resistant monomers, the expansion ratios of the polymer microspheres after 3 days of swelling in deionized water and water with salinities of 5000 mg/L, 15,000 mg/L, and 25,000 mg/L are 4.65, 4.34, 4.05, and 3.58, respectively. Moreover, during long-term swelling experiments, the microspheres maintain comparable expansion ratios with increasing salinity and show no signs of ion-induced rupture. This demonstrates excellent swelling capacity and salt resistance.

(2) The effect of different temperatures on the expansion properties of microspheres

**Figure 13:**
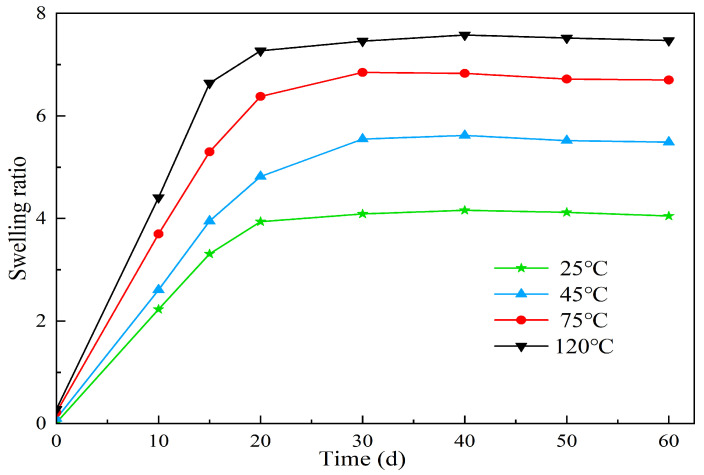
The effect of different temperatures on the swelling properties of polymer microspheres.

Polymer microspheres were prepared in oilfield backflow water with a salinity of 25,000 mg/L. The microspheres were formulated as a 3 wt% solution with a volume of 100 mL. The solution was sealed in vials with blue caps and placed in a thermostatic chamber at temperatures of 25 °C, 75 °C, and 120 °C for a duration of 60 days. Samples were taken periodically to measure the particle size of the microspheres and calculate the swell factor. The experimental results are presented in Table 5 and Figure 13.

According to Figure 13, it can be observed that the swell factor of the polymer microspheres increases with time under the same environmental temperature. Moreover, as the temperature increases, the time taken to reach the peak swell factor also decreases. Under the same aging time conditions, the swell factor of the microspheres increases with higher temperatures. For instance, at 120 °C, the microspheres reach their peak swell factor on the 7th day. Conversely, at lower temperatures, the time taken to reach the peak swell factor is extended. This behavior can be attributed to several factors. Firstly, as the temperature increases, the hydrolysis of hydrophilic groups accelerates, resulting in increased ion activity. This enhances the displacement ability of solvents and polymer molecules, leading to a faster water absorption and swelling rate of the microspheres. Secondly, the intermolecular binding forces between the long chains of the microspheres weaken under the influence of Van der Waals forces at higher temperatures, facilitating the elongation of the polymer chains. As a result, the swell factor of the microspheres increases. In addition, the solvation effect of the solvent on the polymer microball molecular chains gradually increases, and the association between the microballs and water molecules is an endothermic process. Increasing the temperature favors the enhancement of the interaction between the microballs and water molecules.

#### 2.2.4. Evaluation of Stability Performance

**Figure 14:**
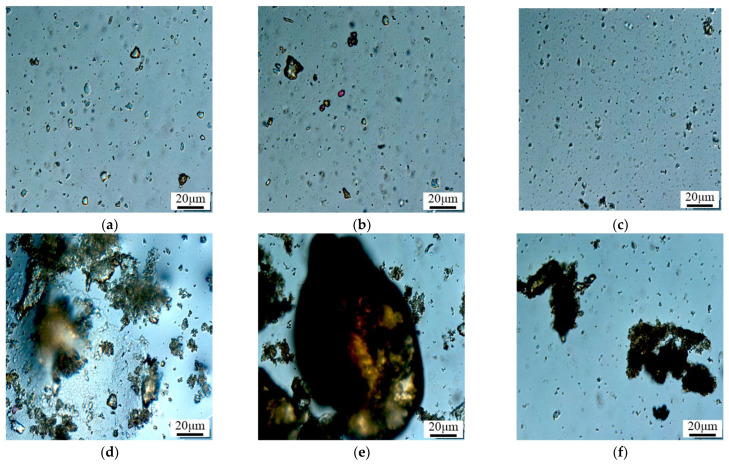
Microscope photograph of long-term stability of microspheres. (**a**) 1 day; (**b**) 3 day; (**c**) 7 day; (**d**) 15 day; (**e**) 60 day; (**f**) 90 day.

The microsphere solutions, aged for different durations, were examined under a microscope, and the results of the aging experiment are shown in Figure 14.

Figure 14 depicts the morphological changes in polymer microspheres (407 nm) after long-term aging, as observed under an electron microscope. The photographic results reveal that the microspheres undergo size expansion to the submicron and micron levels after aging. Agglomeration of the polymer microspheres occurs on the 15th day of aging, and by day 60, the microsphere particles self-aggregate, forming larger clusters exceeding the throat diameter. This effectively improves the water absorption profile and achieves the purpose of profile control and water blocking. In Figure 14f, it can be observed that after 90 days, in a high-temperature and high-salinity environment, some degradation of the microsphere particles occurs, but they still exist in cluster form. This indicates that the microspheres possess good thermal stability.

### 2.3. Evaluation of Profile Control Performance of Polymer Microspheres

#### 2.3.1. Evaluation of Plugging Performance of Microsphere Particles

Through the testing of microsphere particles’ plugging performance in reservoirs with different permeabilities, following the experimental procedure mentioned earlier, a microsphere solution was injected into four groups of sand-packed tubes with varying permeabilities. The prepared polymer microsphere solution was injected into the sand-packed tubes, and the pressure changes at the injection end of the tubes were recorded to plot the pressure curve. The resistance coefficient and plugging efficiency of the sand-packed tubes were calculated using Formulas (2)–(4) as mentioned. This evaluation was performed to assess the plugging effectiveness of the polymer microsphere concentration on the throat and determine the optimal injection volume.

**Figure 15:**
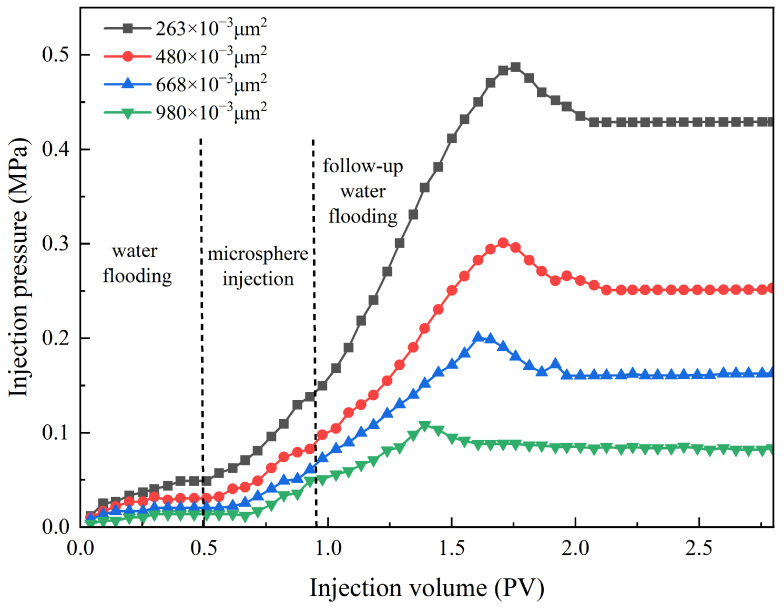
The injection pressure change in polymer microspheres under different permeability.

The displacement flow rate was set at 1.0 mL/min. The environmental temperature was set at 75 °C, with a aging period of 7 days. The salinity of the backflow water was set at 25,000 mg/L, and the concentration of the polymer microsphere solution was set at 3000 mg/L. A volume of 0.3 pore volume (PV) was injected. The pressure curve of the microsphere solution injection is shown in Figure 15. The parameters of the sand-packed tubes are provided in Table 6, and the plugging capacity is presented in Table 7.

From Figure 15, during the microball injection stage, the injection pressure increases as the core permeability decreases. Compared to the water flooding stage, the pressure rise is more pronounced because the injection of the microball solution effectively increases the viscosity of the water phase, thereby enhancing the flow resistance during displacement. In the subsequent water flooding stage, the lower the permeability, the faster the pressure rise, and the higher the breakthrough pressure. Consequently, the final stabilized pressure is also relatively higher. It can be observed that after injecting polymer microsphere particles into sand-packed tubes with different permeabilities, the injection pressure for the secondary water flooding is higher compared to the primary water flooding, indicating the plugging effect of the microspheres. Table 5 shows that the plugging efficiency and resistance coefficient decrease as the permeability increases. When the permeability is 263 × 10^−3^ μm^2^, the residual resistance coefficient reaches 9.09, and the plugging efficiency reaches 89.02%. However, when the permeability increases to 980 × 10^−3^ μm^2^, the residual resistance coefficient drops to only 5.25, and the plugging efficiency decreases to 80.95%. This is because higher permeability corresponds to larger throat radii. Although the polymer microspheres can easily enter the pore interiors, they struggle to aggregate and plug at the throats. Consequently, their retention in the formation is lower, resulting in reduced plugging efficiency. Therefore, for reservoirs with higher permeability, the injection of polymer microsphere particles exhibits better fluidity, but the plugging effect is compromised.

#### 2.3.2. Evaluation of the Effect of Double-Tube Parallel Flow Steering of Polymer Microsphere Particles

**Figure 16:**
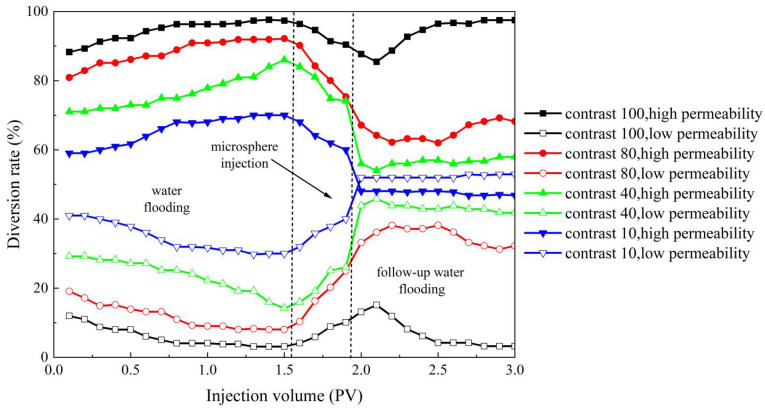
High- and low-permeability diversion rate curves under different permeability differential conditions.

According to the experimental method described earlier for improving the profile performance, this section designs four sets of experimental models with different permeability differentials. The permeability differentials for the four sets are 100, 80, 40, and 10, respectively. The water diversion rate curves in Figure 16 provide a visual representation of the changes in water absorption profiles of the core samples during the three stages: primary water flooding, microsphere injection, and subsequent water flooding. When the permeability differential between high- and low-permeability cores is 100, the differential is too large, resulting in the microspheres being unable to improve the water absorption profile effectively. However, as the permeability differential decreases, the microspheres reach their aging state, and the liquid flow rate through the high-permeability core gradually decreases, while the flow rate through the low-permeability core gradually increases. This leads to effective improvement in the water absorption profile of the high- and low-permeability cores. In terms of profile improvement rate, the most significant improvement is observed when the permeability differential is 80, with a profile improvement rate of 81.5%. Conversely, the lowest profile improvement rate is observed when the permeability differential is 10, with a rate of 62%. This can be attributed to the lower permeability differential, where the permeabilities of the high- and low-permeability cores are closer. During the primary water flooding stage, the ratio of the water diversion rates between high- and low-permeability cores is already significantly lower compared to other permeability differentials. Based on these observations, it can be concluded that when the permeability differential is too large, specifically exceeding 100, the microspheres are unable to effectively improve the water absorption profile. However, within a certain range of permeability differentials, specifically between 10 and 80, the microspheres can effectively improve the water absorption profile. Moreover, within this range, a larger permeability differential results in a better improvement effect on the water absorption profile.

#### 2.3.3. Evaluation of the Enhanced Oil Recovery Effect of Polymer Microsphere Particles

**Figure 17:**
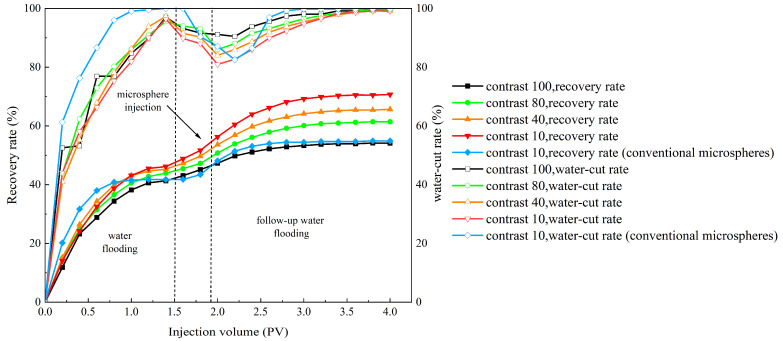
The changes in the recovery rate and water-cut rate during the displacement process of self-cementing polymer microspheres and conventional microspheres with varying injection volumes.

Based on the experimental methods for evaluating oil displacement effectiveness described above, this section designs four experimental models with different permeability differentials: 100, 80, 40, and 10. According to Table 8 and Figure 17, it can be seen that the oil displacement effect can corroborate the profile improvement effect. At a permeability differential of 100, the poor plugging effect results in a limited increase in subsequent water flooding recovery, with a final recovery rate of only 54.1%. As the permeability differential decreases, the self-aggregating polymer microballs exhibit their unique self-aggregating properties, coupled with improved plugging effects, leading to a significant increase in both low-permeability layer recovery and total recovery. At permeability differentials of 80, 40, and 10, the recovery rates increased by 17.5%, 20.3%, and 24.3%, respectively. This indicates that within the permeability differential range of 10 to 80, the oil displacement effect is significantly enhanced by the microball solution. Compared with ordinary polymer microballs, the injection of self-aggregating polymer microballs increased the final recovery rate by 15.6%, whereas NM microballs only increased it by 13.3%. Therefore, the self-aggregating polymer microballs demonstrate a substantially improved oil displacement effect compared to ordinary polymer microballs.

#### 2.3.4. Evaluation of Polymer Microsphere Particles Microporous Throat Migration-Plugging Oil Displacement Performance

By employing micro-scale etched glass models for oil displacement experiments, we investigated the transport, retention, and deformation characteristics of gel systems within porous media, as illustrated in Figure 17.

**Figure 18:**
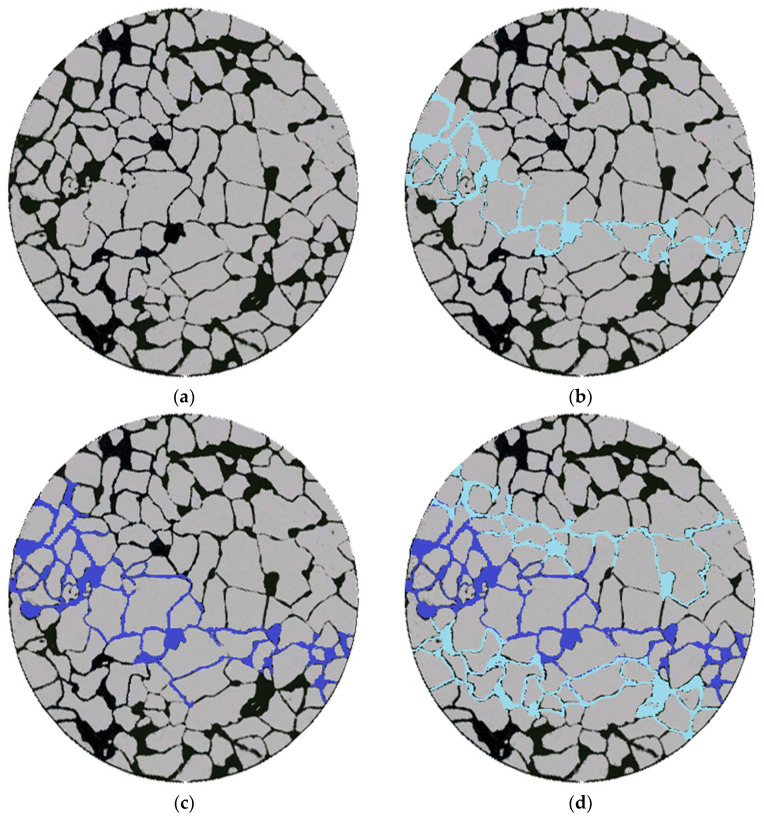
Microscopic simulation of glass etching model oil displacement effect diagram. (**a**) Initial state; (**b**) water flooding; (**c**) microsphere injection; (**d**) follow-up water flooding.

After the micro-scale displacement by water flooding, the water flow primarily follows the main flow pathways, resulting in fingering phenomena. The regions outside the main flow pathways contain a significant amount of remaining oil, with a lower degree of depletion and a smaller affected area. Approximately 67.2% of the original oil remains untapped in these regions. Upon injecting the polymer microspheres into the model (Figure 18c), the microspheres enter the larger channels and move forward under pressure. This leads to further depletion of the oil, increasing the affected area to 40.7%. After the microsphere injection process, subsequent water flooding is conducted (Figure 18d). The microspheres plug the original large channels, causing the water flooding to change its flow direction. This displacement affects the non-main flow pathways and the low-permeability regions, resulting in enhanced oil recovery and an expanded affected area. The overall oil recovery reaches 48.2%, representing a 33.7% increase in recovery compared to the previous stages. This demonstrates the effectiveness of improving the oil recovery rate through the expansion of the affected area.

## 3. Materials and Methods

### 3.1. Reagents and Instruments

Experimental reagents: Acrylamide (AM, crystal, ≥98%), acrylic acid (AA), sourced from Jiangxi Jiujiang Chemical Company Jiujiang City, China; 2-acrylamido-2-methylpropanesulfonic acid (AMPS), polyethylene glycol diacrylate (PEGDA), sourced from Aladdin Biochemical Technology Co., Ltd., Shanghai, China; N,N′-methylenebisacrylamide (MBA), sodium hydroxide (NaOH), ethanol (C_2_H_6_O), propanol (C_3_H_6_O), ammonium persulfate (APS), sodium bisulfite (SHS), SPAN80, TWEEN80, sourced from Chengdu Kelong Reagent Factory, Chengdu, China.

Properties of the water used: Produced water from the oilfield with a total dissolved solids (TDS) of 25,396.52 mg/L and ion concentrations (mg/L) as follows: Ca^2+^ 999.70, Mg^2+^ 163.35, CO_3_^2−^ 32.91, HCO_3_^−^ 407.19, Cl^−^ 14,496.6, SO_4_^2−^ 785.05, Fe^3+^ 0.79, Al^3+^ 0.15, K^+^ 51.72, Na^+^ 8459.06.

Experimental equipment: Electric blast constant temperature drying oven, Huitai Instrument Manufacturing Co., Ltd., Shanghai, China; QuantaFEG 450 type Environmental Scanning Electron Microscope (SEM), FEI Company, Hillsboro, FL, USA; Nicolet iS10 type infrared spectrometer, Thermo Nicolet Corporation, Madison, IL, USA; HBYQ-2 high-temperature and high-pressure core flow test device, Huabao Petroleum Instrument Co., Ltd., Yangzhou, China; HBS300/50 double cylin-der constant speed constant pressure pump, Huabao Petroleum Instrument Co., Ltd., Yangzhou, China; Microsimulation glass etching model, homemade; OLYMPUS BX41 type op-tical microscope, microscope, Olympus Corporation, Tokyo, Japan; Micropump, Hai ‘an Petroleum Scientific Research Instrument Co., Ltd., Nantong, China; ZN-08 type laboratory shredder, Xingshi Lihe Technology Development Co., Ltd., Beijing, China; DF-101S collector constant temperature heating magnetic stirrer, Gongyi Kehua Instrument Co., Ltd., Gongyi, China; Hoffen-10 Fourier transform infrared spectrometer, German company Bruker, Saarbrücken, Germany; TGA-601 Thermogravimetric Analyzer, TA Instruments, Newcastle, DE, USA; NS-90Z Nanoparticle Size and Potentiometric Laser Particle Sizer, Zhuhai Euclid Instrument Co., Ltd., Zhuhai, China; LC-LX-H185C Desktop High Speed Centrifuge, Shanghai Lichen Instrument Technology Co., Ltd., Shanghai, China.

### 3.2. Experimental Methods

(1) Preparation of polymer microspheres: The three-component polymer microspheres, namely AM-AA-AMPS, were prepared using a reverse microemulsion polymerization method. Deionized water was selected as the aqueous phase, while paraffin oil was chosen as the oil phase. Ammonium persulfate, a water-soluble oxidizing agent, and sodium bisulfite, a reducing agent, were used as the initiators. N,N′-methylenebisacrylamide and polyethylene glycol diacrylate were used as the crosslinking agents. By incorporating the emulsifiers Span80 and Tween80, the reverse microemulsion polymerization method was employed to obtain nanoscale polymer microspheres with temperature and salt resistance, as well as delayed swelling characteristics.
(2) Infrared spectroscopic characterization of polymer microspheres: The infrared characterization of the microspheres was performed using the potassium bromide (KBr) pellet method. A small amount of dry microsphere powder was mixed and ground with a small amount of KBr powder to obtain a fine powder. The mixture was uniformly spread in a mold, and pressure was applied to produce transparent thin pellets. These pellets were then placed on the sample holder of a Bruker infrared spectrometer for scanning. The scanning range was set between 400 and 4000 cm^−1^, and the infrared absorption spectra were measured and recorded [43].
(3) Scanning electron microscopy evaluation of polymer microspheres: A small amount of dry microsphere powder was evenly dispersed onto a conductive adhesive attached to the sample stage to prepare the sample. The sample was then coated with a thin layer of gold using an ion sputtering device to prevent charge accumulation and ensure accurate results. The micro-scale morphology of the polymer microspheres was observed using a scanning electron microscope (SEM), and micrographs of the dry microspheres were captured at different magnifications, documenting their microscopic shape and structure [44,45].
(4) Temperature Resistance Evaluation of Polymer Microspheres: A small amount of dry polymer microspheres is taken, and thermogravimetric analysis (TGA) is performed on the polymer microsphere samples using a TGA instrument. The temperature range is set from 0 to 800 °C, with a heating rate of 10 °C/min, under a nitrogen atmosphere. The sample is heated from room temperature to 800 °C, and the generated data are exported to generate a data curve for the temperature resistance evaluation of the polymer [46,47,48].
(5) Evaluation of salt resistance of polymer microspheres: A certain amount of the prepared polymer microsphere dry powder was placed in deionized water and different salinity produced water samples. Polymer microsphere solutions were prepared by mixing the microsphere powder with the respective water samples. The solutions were then placed in a thermostatic chamber set at 25 °C. Samples were taken at intervals of 0 days, 3 days, 15 days, and 30 days. Each time, a small amount of the test solution was placed in an ultrasonic bath for dispersion. The dispersed solution was then transferred into a measuring tube, and the median particle size was measured using a particle size analyzer.
(6) Evaluation of the swelling behavior of polymer microspheres: Experimental data on the influence of microsphere emulsion particle size under different salinity levels are collected. The swelling factor of the microspheres is calculated using Equation (1) to demonstrate the effect of salinity on the swelling capacity of the microspheres. Temperature is then introduced as a factor, and the particle size changes before and after swelling are measured using a laser particle size analyzer. A comparison is made between the prepared microspheres to evaluate their swelling performance.

According to the formula for the aging swelling factor of microspheres, the expansion factor Q of the microspheres is calculated using the following equation:

(1) $$Q=\frac{D_{2}-D_{1}}{D_{1}}\times 100\%$$

in the equation: *D*_1_ represents the initial median particle size of the microspheres, in nanometers (nm); *D*_2_ represents the median particle size of the microspheres after expansion, in nanometers (nm).

(1) Evaluation of the stability of polymer microspheres: A 100 mL volume of a 3 wt% microsphere solution was prepared using oilfield produced water with a salinity of 25,000 mg/L. The solution was transferred into a blue-capped reagent bottle and sealed. The bottle was then placed in a thermostatic chamber for aging, with the temperature set at 120 °C. At regular intervals, a portion of the microsphere solution was taken out and observed under an optical microscope at a magnification of 500×. The morphological characteristics of the microsphere particles were observed and recorded [49,50].
(2) Evaluation of the plugging performance of polymer microspheres: Different formulations of polymer microsphere solutions were prepared for core flow experiments. A Φ3.8 cm × 100 cm sand-packed tube with four pressure measuring points was used. The pressure data at each measuring point were recorded during the water flooding, injection of polymer microsphere solution, and subsequent water flooding processes. The plugging efficiency of the polymer microspheres was calculated based on the pressure data [51].

The formulas for calculating the resistance coefficient, residual resistance coefficient, and plugging efficiency are shown in Equations (2)–(4):

(2) $$F_{R}=\frac{\Delta P_{2}}{\Delta P_{1}}$$

(3) $$F_{RR}=\frac{\Delta P_{3}}{\Delta P_{1}}$$

(4) $$\eta =\frac{\Delta P_{3}-\Delta P_{1}}{\Delta P_{3}}$$

in the equations: *F_R_* represents the resistance coefficient, a dimensionless quantity; *F_RR_* represents the residual resistance coefficient, a dimensionless quantity; *η* represents the plugging efficiency (%); Δ*P*_1_ represents the pressure difference at the sand-packed tube inlet during water flooding (MPa); Δ*P*_2_ represents the pressure difference at the sand-packed tube inlet during microsphere inject ion (MPa); Δ*P*_3_ represents the pressure difference at the sand-packed tube inlet during secondary water flooding (MPa).

(1) Study of fluid flow diversion using dual-tube parallel flow with polymer microspheres: Dual-tube parallel oil displacement experiments were conducted using artificial cores with different permeabilities (Φ2.5 cm × 10 cm). The changes in effluent volume and pressure before and after plugging were recorded for both high- and low-permeability cores [28,52].
(2) Study on the Enhanced Oil Recovery Effect of Polymer Microspheres: Dual-tube parallel oil displacement experiments were conducted using artificial cores with different permeabilities (Φ2.5 cm × 10 cm). Record the effluent volume and oil production at the outlet end of high- and low-permeability cores. Calculate the comprehensive water-cut and recovery factor of microspheres under different permeability gradients.
(3) Study of the migration-plugging characteristics of polymer microspheres in microscopic pore throats: A saturated microfluidic glass etching model was prepared. The model was subjected to water flooding, injection of stained polymer microspheres (with methylene blue), and subsequent water flooding. Images of the model were processed using Image J software (the version number of this software is 1.54g) to study and analyze the microscopic distribution characteristics of oil and water during different displacement stages [53,54].

## 4. Conclusions

(1) Temperature- and salt-resistant self-curing polymeric microspheres were synthesized by introducing temperature- and salt-resistant monomer AMPS and hydrophobic chains into a copolymerization reaction. The microspheres were synthesized using a reverse microemulsion polymerization method, resulting in nanoscale particles with regular spherical morphology.
(2) The nanoscale polymeric microspheres exhibit temperature and salt resistance. Partial thermal weight loss was observed for the microspheres at temperatures as high as 295 °C. Under high-salinity conditions, the microspheres maintain a significant expansion ratio, reaching up to 4.17 times their original size. The expansion of the microspheres is facilitated by the strong hydrophilic groups present on the polymer chains, which interact with water. Moreover, the three-dimensional network structure of the microspheres, combined with internal entanglement, generates osmotic pressure that promotes water absorption and expansion.
(3) Under high-temperature and high-salinity conditions, the polymeric microspheres exhibit agglomeration after 15 days of aging. However, even after 90 days of aging, they maintain a certain degree of self-curing. No significant hydrolysis occurs, indicating that the polymeric microspheres possess long-term stability.
(4) The plugging efficiency, resistance coefficient, and residual resistance coefficient of the polymeric microspheres decrease with an increase in reservoir permeability. For a permeability of 980 × 10^−3^ μm^2^, the plugging efficiency is only 80.95%, while at a permeability of 263 × 10^−3^ μm^2^, the plugging efficiency reaches 89.02%. The nanoscale microspheres exhibit excellent plugging performance in low-permeability reservoirs.
(5) Oil displacement and profile improvement effects mutually corroborate. When the permeability differential is 100, the plugging effect is poor, resulting in a recovery rate increase to only 54.1%. As the differential decreases, self-aggregating polymer microballs exhibit unique self-aggregation capabilities, significantly enhancing recovery rates in low-permeability layers and overall. The recovery rate increases at different differentials of 80, 40, and 10 are 17.5%, 20.3%, and 24.5%, respectively. Within the range of differentials from 10 to 80, oil displacement effectiveness significantly improves. Compared to conventional microballs, self-aggregating polymer microballs increase final recovery rates by 13.3%, marking a substantial improvement.
(6) The polymeric microspheres plug the main flow channels with high permeability. Microscopic etched glass displacement experiments demonstrate that after the injection of polymeric microspheres, a flow diversion occurs, expanding the affected volume in the medium-to-low-permeability and non-mainstream regions. This leads to a 33.7% increase in oil recovery.

## Figures and Tables

**Table 1:** Analysis of infrared characteristic groups of dispersed plugging agent.

Absorption Peak/cm^−1^	Corresponding Group (Stretching Vibration)
3345	The N-H bond on -CONH
2965	-CH_2_-
2842	-CH_3_
1781, 1542	-C=O
1219, 1119, 1033	-SO_3_^−^

**Table 2:** Observation of microspheres under different dispersants.

Blocking Agent	Reinjection Water	Anhydrous Ethanol	Kerosene
nanoscale microsphere granule	there is agglomeration phenomenont	obvious reunion, and agglomerated particles are large	large-scale agglomeration

**Table 3:** The median particle size parameters of polymer microspheres under different salinity.

Mineralization/mg	Swelling Time/d			
	0	3	15	30
0	0.51 μm	2.61 μm	2.92 μm	2.92 μm
5000	0.49 μm	2.09 μm	2.56 μm	2.56 μm
15,000	0.44 μm	1.93 μm	2.22 μm	2.26 μm
25,000	0.41 μm	1.75 μm	2.06 μm	2.10 μm

**Table 4:** Microsphere expansion results under different salinity.

Mineralization/mg	Expansion Time/d							
	0	10	15	20	30	40	50	60
0	0.53 μm	2.16 μm	2.86 μm	3.15 μm	3.27 μm	3.26 μm	3.25 μm	3.23 μm
5000	0.50 μm	1.76 μm	2.12 μm	2.45 μm	2.65 μm	2.64 μm	2.63 μm	2.61 μm
15,000	0.45 μm	1.30 μm	1.69 μm	1.91 μm	2.00 μm	2.03 μm	2.01 μm	1.97 μm
25,000	0.41 μm	1.05 μm	1.35 μm	1.62 μm	1.66 μm	1.71 μm	1.67 μm	1.62 μm

**Table 5:** Microsphere expansion results under different temperatures.

Temperatures/°C	Expansion Time/d							
	0	10	15	20	30	40	50	60
25	0.45 μm	1.01 μm	1.49 μm	1.77 μm	1.84 μm	1.87 μm	1.85 μm	1.82 μm
45	0.48 μm	1.25 μm	1.90 μm	2.31 μm	2.66 μm	2.70 μm	2.65 μm	2.62 μm
75	0.51 μm	1.89 μm	2.70 μm	3.25 μm	3.49 μm	3.48 μm	3.42 μm	3.40 μm
120	0.54 μm	2.38 μm	3.59 μm	3.93 μm	4.03 μm	4.09 μm	4.06 μm	3.99 μm

**Table 6:** Sand filling pipe parameter table.

Numbering	Length/cm	Inside Diameter/cm	Porosity/%	Permeability/10^−3^ μm^2^
S-1	100	3.8	20.1	210
S-2			25.5	507
S-3			28.6	712
S-4			33.2	1106

**Table 7:** The influence of different permeability on the plugging performance of microsphere system.

Permeability/×10^−3^ μm^2^	Resistance Coefficient	Residual Resistance Coefficient	Plugging Efficiency/%
263	2.77	9.09	89.02
480	2.60	8.17	87.80
683	2.44	7.27	86.31
980	2.31	5.25	80.95

**Table 8:** Experimental results of oil displacement.

Permeability/×10^−3^ μm^2^		Contrast	Types of Microspheres	Recovery Rate/%	
High Permeability	Low Permeability			Before Injecting Microspheres	After Injecting Microspheres
1000	10	100	Self-cementing nanoscale polymeric microspheres	41.3	54.1
800	10	80		43.9	61.4
800	20	40		45.3	65.6
200	20	10		46.2	70.7
200	20	10	Conventional microspheres	41.8	55.1

## Data Availability

All relevant data have been presented in this paper.
